# Supplementary material for: Microstructural Study of Two-Dimensional Organic-Inorganic Hybrid Perovskite Nanosheet Degradation under Illumination
Source: Nanomaterials (Basel). 2019 May 10;9(5):722. doi: 10.3390/nano9050722 (PMC6566729; doi:10.3390/nano9050722)
Supplement: Supplementary file 1 [file nanomaterials-09-00722-s001.pdf]

nanomaterials

Supplementary Material

Microstructural Study of Two-Dimensional Organic-Inorganic Hybrid Perovskite Nanosheet Degradation under Illumination

Lingfang Nie, Xiaoxing Ke * and Manling Sui

Beijing Key Lab of Microstructure and Property of Advanced Solid Material, Institute of Microstructure and Properties of Advanced Materials, Beijing University of Technology, Beijing 100124, China; nielingfang1210@163.com (L.N.); mlsui@bjut.edu.cn (M.S.)

* Correspondence: kexiaoxing@bjut.edu.cn

HRTEM was performed for 2D perovskite nanosheets. We used a FEI Titan G^2 microscope operated at 300 kV. Spot size 6 was used in order to minimize damage as much as possible.

Figure S1 demonstrates nanosheets acquired at different magnifications. It can be seen that even at low magnification where the dose rate is kept minimum, nanoparticles precipitate almost immediately when the electron beam is on the sample and the nanosheets are damaged. The corresponding fast Fourier transform (FFT) patterns confirm the structure to be completely amorphized.

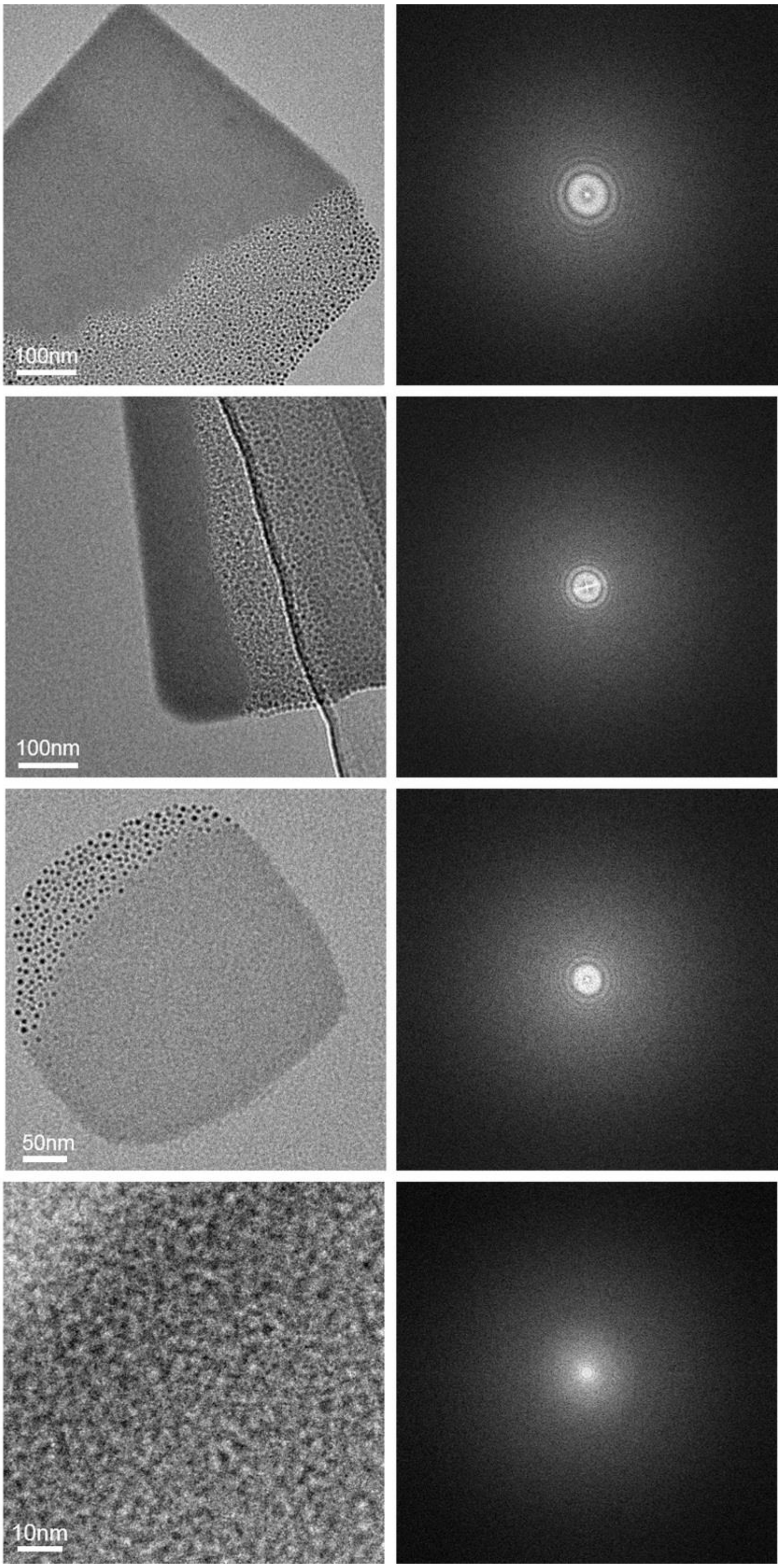

Figure S1. TEM images and the corresponding fast Fourier transform (FFT) patterns of BA_2PbBr_4 nanosheets at different magnifications.

Figure S2 demonstrates a time series of a nanosheet during acquisition. It is clearly seen from the image series that nanoparticles precipitate almost immediately once exposed to electron beam, and such precipitation quickly expands through the entire nanosheet. Corresponding FFT patterns

show that the nanosheets are amorphized, demonstrating rapid deterioration of nanosheets during imaging.

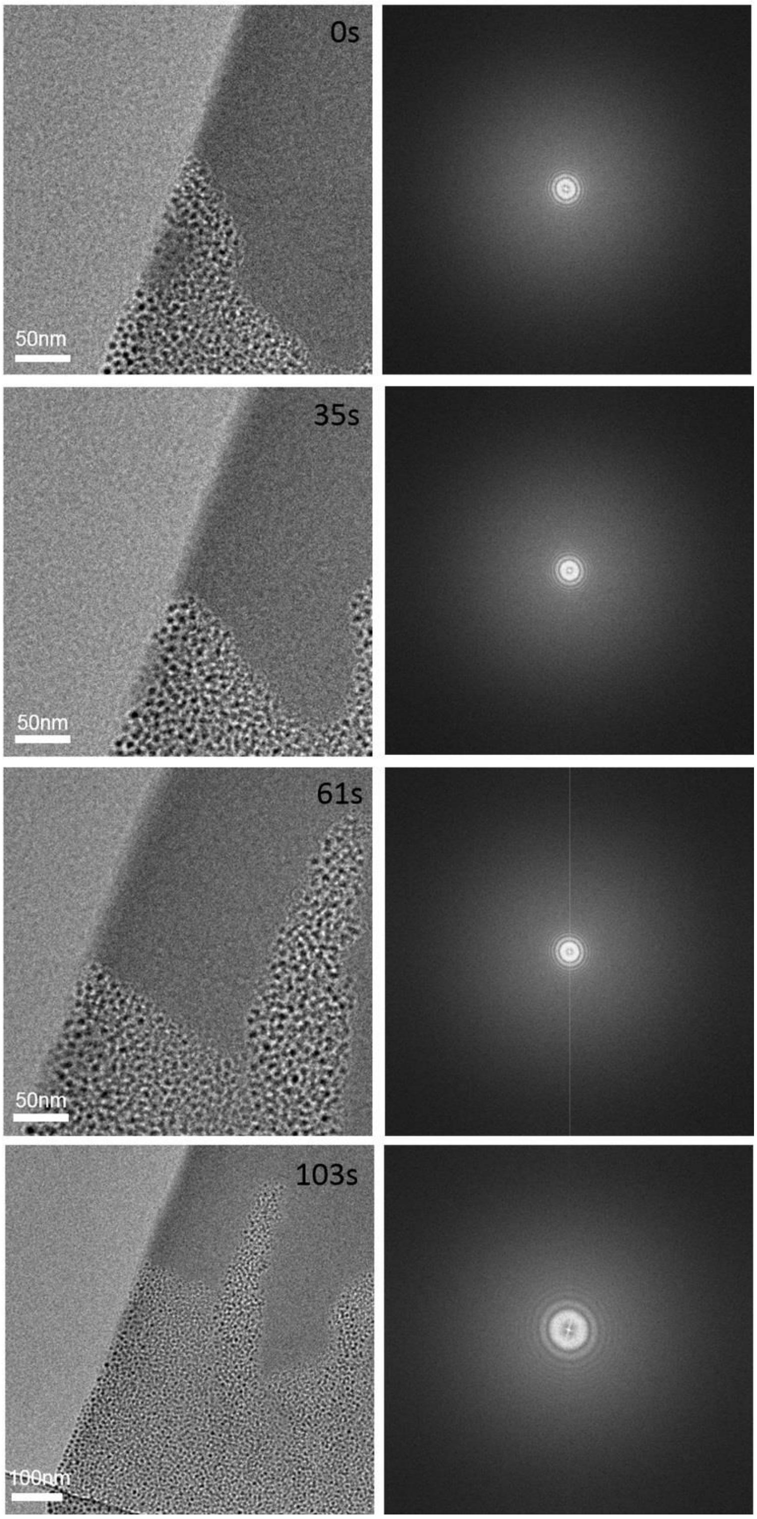

Figure S2. TEM images and the corresponding FFT patterns of a BA_2PbBr_4 nanosheet under electron beam during imaging.
